# Development of a Spatial Discount Task to Measure Impulsive Choices in Dogs

**DOI:** 10.3390/ani9070469

**Published:** 2019-07-23

**Authors:** Paolo Mongillo, Anna Scandurra, Carla Jade Eatherington, Biagio D’Aniello, Lieta Marinelli

**Affiliations:** 1 Laboratorio di Etologia Applicata, Dipartimento di Biomedicina Comparata e Alimentazione, Università degli Studi di Padova. Viale dell’Università 16, 35020 Legnaro, Italy; 2 Dipartimento di Biologia, Università di Napoli Federico II, Via Cinthia, Edificio 7, 80126 Napoli, Italy

**Keywords:** dog, behavioral test, impulsivity, sex differences, learning, validation

## Abstract

**Simple Summary:**

Impulsivity is believed to play a role in problematic behaviors in dogs. In this study, we developed a test to assess dogs’ tendency to make impulsive choices, that is their preference for smaller immediate reward instead of larger, but harder to obtain ones. Dogs were first trained that a bowl presented on a certain side always contained a large food amount, whereas the one presented on the opposite side (although at the same distance from the dog) contained less food. Then, the bowl with less food was progressively placed closer to the dog. As expected, dogs’ choices to feed from the bowl with less food increased as the distance of the latter decreased. Choices did not depend on factors that could interfere, such as dogs’ level of motivation for food, training experience, or learning ability. This indicates that the test is likely to be actually assessing impulsivity, not other traits. Also, female dogs were more likely to make impulsive choices than males, in accordance with what is known in humans and rodents, supporting the validity of the test. The test was completed in less than 1 h, making it a valid option to assess impulsivity in dogs in various contexts.

**Abstract:**

Impulsive choices reflect an individual’s tendency to prefer a smaller immediate reward over a larger delayed one. Here, we have developed a behavioural test which can be easily applied to assess impulsive choices in dogs. Dogs were trained to associate one of two equidistant locations with a larger food amount when a smaller amount was presented in the other location, then the smaller amount was placed systematically closer to the dog. Choices of the smaller amount, as a function of distance, were considered a measure of the dog’s tendency to make impulsive choices. All dogs (N = 48) passed the learning phase and completed the entire assessment in under 1 h. Choice of the smaller food amount increased as this was placed closer to the dog. Choices were independent from food motivation, past training, and speed of learning the training phase; supporting the specificity of the procedure. Females showed a higher probability of making impulsive choices, in agreement with analogue sex differences found in human and rodent studies, and supporting the external validity of our assessment. Overall, the findings support the practical applicability and represent a first indication of the validity of this method, making it suitable for investigations into impulsivity in dogs.

## 1. Introduction

Impulsivity is generally referred to as the tendency to act prematurely, without forethought or consideration of the consequences [1], or as the failure to defer gratification [2]. In humans, impulsivity has been indicated as a vulnerability factor for a range of maladaptive behaviours, including substance abuse, gambling, or pathological conditions such as attention deficit and hyperactivity disorders [3,4]. Although impulsivity is sometimes measured as a single dimension of personality, it is best described as a multidimensional trait [4]. Many studies converge on the recognition of two broad classes of impulsive behaviour, namely impulsive actions and impulsive choices [5,6]. The former is regarded as the result of an inability to inhibit or stop a motor act in response to prepotent stimuli. Behavioural paradigms such as the go/no-go task, or the stop-signal reaction time task, analogue versions of which exist for humans and rodents, are designed to pinpoint this behavioural facet of impulsivity [7]. Impulsive choices instead reflect an individual’s preference for smaller immediate gratifications over delayed ones of greater value or quantity [8]. This dimension of impulsive behaviour is typically assessed in delay-discounting tasks, which measure the maximum delay tolerated by individuals who are informed of (for humans) or trained to expect (for animal paradigms) the possibility to obtain higher value rewards if they can wait for sufficiently long time-intervals [7,9]. These tasks do not simply represent different measures of a single construct. There is evidence that these two measures are independent [10,11], and that they are underpinned by different neurobiological mechanisms [12,13,14]. Also, they are differently related to individual characteristics, such as sex and age. For instance, while a tendency to perform impulsive actions is blandly, if at all, associated with male sex, robust associations exist between sex and impulsive choices, where females discount more steeply than males in both humans and rodents [15]. Sex differences are believed to root in differential activation of the dopaminergic signalling system between sexes, which mediate subjects’ sensitivity to rewards. Less clear is the interplay between these mechanisms and circulating gonadal hormones, the role of which impulsive choice behaviour still has to be clarified [15]. As regards age, evidence indicates a higher tendency to express impulsive choices during adolescence/young adulthood, than later in life [16].

The current knowledge about impulsivity comes mostly from studies in humans and rodents. However, the same construct has been tentatively applied to dogs, where high impulsivity is thought to be a correlate of different maladaptive behavioural manifestations or cognitive processes. For instance, impulsivity may play a role in aggression [17,18], and more generally in the expression of behavioural problems [19]. Furthermore, some evidence suggests that impulsivity is associated with lower problem-solving abilities [20,21]. As it occurs in the human literature, methods used to assess impulsivity in dogs vary in scope and methodology. A plethora of tasks that were proposed as assessments of dogs’ impulsivity actually represent (tentative) measures of impulsive actions, including reversal learning tasks, the A-not-B task, the cylinder task the middle-cup task, the wait-for-treat task, and buzzer tasks [21,22,23,24,25,26]; although a thorough description of these paradigms fall outside the scope of this paper, all encompass the necessity to withhold a prepotent response, either spontaneous or learnt. A much smaller variety of tasks assess impulsive choices. Although with some variations in the nature or the source (social or non-social) of the reward, these methods are based on the same general paradigms which measure dogs’ ability to tolerate temporal delays on the expectation of a larger/more valuable reward [17,25,27,28,29]. A common disadvantage of these delay-discounting tasks is that they generally require dogs to undergo a long training (in most cases lasting more than one day), which also makes it difficult to complete the test as proved by a relatively low success rate (e.g., 58.8% [19]; 51.4% [30]). This obviously represents a strong limit to the practical applicability of these tasks, and to the possibility of administering them routinely to large dog samples.

There is accumulating evidence that measures provided by these methods are in many cases uncorrelated [23,30]. Moreover, there is variability in terms of how the outcomes of these tasks relate to the broader, indirect assessment of impulsivity provided by owners’ answers to a questionnaire (Dog Impulsivity Assessment Scale, DIAS [18]), which range from no correlation [29,30], to correlation with one of the DIAS subscales [19] or with the overall DIAS score [19,30]. Although this lack of consistency may reflect the complex, multidimensional nature of the construct, it nonetheless prompts us to question which of these tasks provide a valid and easy measure of impulsive behaviours in dogs. In only a few cases, attempts have been made to assess impulsivity as a function of external variables known to influence impulsivity measures in other species, such as sex or age (see for instance [30]). However, none of the aforementioned studies addressed problems of potential intervening variables, including dog’s motivation for food, learning abilities, and previous experience, which may represent confounds in the outcome of the assessments, as highlighted by some of the very same authors [30].

Upon these premises, in this study we aimed to develop and validate a task to assess dogs’ tendency to express impulsive choices. In view of the possibility to administer the task to large dog samples, one of the requisites of the task was to be successfully easily completed by most dogs, and within a reasonably short time (i.e., a single session, no longer than 1.5 h). To circumvent the difficulties associated with training the dogs to wait in the classical delay-discounting tasks, here the immediacy of the possibility to obtain the smaller reward was operationalized as a smaller space to travel, rather than as a shorter time to wait (although space differences inherently imply a time difference [31]). In the lack of a gold standard that could provide an external validation measure, we aimed at providing a first assessment of the tasks’ validity, by (a) looking at the psychometric relationship between the task contingencies and dogs’ performance, (b) excluding effects of other intervening factors, namely the dogs’ previous training history, level of food motivation, and the learning requirements of the task, (c) assessing the tasks sensitivity to biological factors that are known to influence impulsive choices in other species, namely sex, reproductive status and age, and (d) looking at the relationship between the outcomes of the task and indirect measures of impulsivity provided by the DIAS questionnaire.

During the writing of this paper, results of the development of an analogous paradigm, independently developed by Brady and collaborators [32], came to our attention. Like the method described in the present paper, the task was a spatial version of the classical delay-discounting task. The procedure involved a single test session, preceded a short pre-training phase, and was completed by dogs in one day. The short time requirement, and a training success rate of 96% (24 out of 25 dogs), provide excellent indications in terms of feasibility of this kind of procedure. As far as validation was concerned, the primary means of validation reported in the study were the assessment of test-retest reliability and correlations with a score of the DIAS. On the other hand, the study did not look at factors included in our investigation, and highlighted by the very same authors as potential confounds in their results. In this sense, the results reported in the present study represent fundamental additional indications about the validity of this spatial-discounting task.

## 2. Materials and Methods

### 2.1. Subjects

Forty-eight pet dogs were recruited for this study through advertisement in veterinary clinics and the University of Padua. Apart from being healthy, no specific criteria for inclusion in the study were required. The sample included 15 mongrels of small (≤30 cm at the withers, N = 2), medium (>30 and ≤55 cm; N = 9) and large size (>55 cm; N = 4), and 33 pure breed dogs (N = 7 Border Collies, N = 4 Australian Shepherds, N = 3 Golden Retrievers, N = 2 Beagles, N = 2 Cocker Spaniels, N = 2 Labrador Retrievers, N = 1 American Staffordshire Terrier, N = 1 Bernese Mountain Dog, N = 1 Breton, N = 1 Czechoslovakian Wolfdog, N = 1 Dachshund, N = 1 German Shepherd, N = 1 Greyhound, N = 1 Hovawart, N = 1 Labradoodle, N = 1 Newfoundland, N = 1 Rhodesian Ridgeback, N = 1 Samoyed, N = 1 Siberian Husky). Recruitment was aimed at forming four groups of equal size based on the dogs’ sex and reproductive status, namely: non-orchiectomized males (mean age ± SD: 4.4 ± 3.2 years, min = 1, max = 12), non-ovariectomized females in dioestrous or anoestrous phase (mean age ± SD: 4.7 ± 2.7 years, min = 1.5, max = 11), orchiectomized males (mean age ± SD: 4.8 ± 2.3 years, min = 1, max = 10) and ovariectomized females (mean age ± SD: 4.8 ± 1.5 years, min = 2, max = 9). Dogs of the last two groups had their gonads removed at least 6 months prior to participating in the study. The owners were asked to indicate if their dogs had any previous experience of training, choosing between four options (no training, basic training with no professional support, obedience training with a professional trainer, training to specific activities with a professional trainer). Finally, owners were asked to evaluate their dog’s food motivation, as high (would always eat if given the chance, eats most types of food, never leaves food in the bowl, fights for food), medium (sometimes leaves food in the bowl, eats many, but not all types of food, does not fight for food), or low (always leaves some food in the bowl, only eats some specific types of food, never fights for food). The distribution of training history and food motivation within each of the four experimental groups is reported in Table 1. Owners were asked to not feed their dogs on the day of the experiment.

### 2.2. Impulsivity Evaluation Questionnaire

Owners were asked to fill out an Italian translation of the DIAS. This required owners to indicate their degree of agreement with the proposed statements, according to a score scale from 1 (complete disagreement) to 5 (complete agreement). For each dog, an Overall Questionnaire Score (OQS) was calculated as the average score obtained in all items. Moreover, for the sake of comparison with other studies, average scores were calculated for three sub-scales corresponding, in terms of item composition, to the three factors described by Wright and collaborators [18]. However, a factor analysis performed on the data collected in the current study resulted in a very different factorial structure (data not reported), thus the sub-scales used in this study could not be described using the same names adopted elsewhere.

### 2.3. Experimental Setting

**Figure 1:**
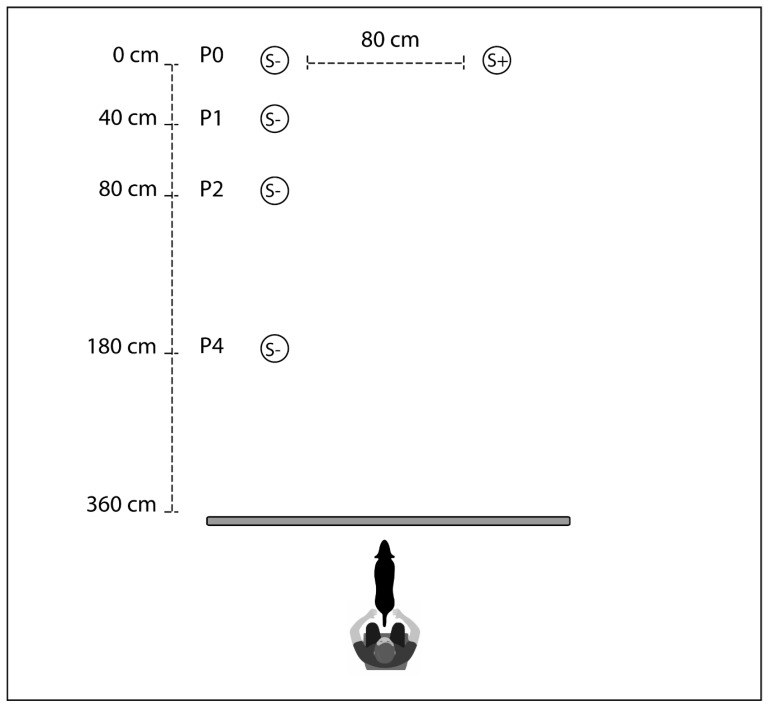
Experimental setting. Representation of the experimental setting, illustrating the owner’s and dog’s position behind the curtain (large horizontal grey bar) at the start of presentations, and the position of the bowls containing the larger (S+) and the smaller amount of food (S−) during training (P0) and test trials (P0 for S+, P0 to P4 for S−).

Tests were conducted at the Laboratory of Applied Ethology (Department of Comparative Biomedicine and Food Science, University of Padua) in a room of approximatively 5 × 5 m, equipped with a chair behind a curtain (140 cm high and 160 cm wide) and two plastic panels (24 × 38 cm), placed vertically at a maximum distance of 360 cm from the chair (the actual distance depended on the experimental phase, as detailed below) and 80 cm apart (Figure 1). The panels represented placeholders for positioning food bowls (circular metal bowls, 20 cm in diameter) during the experiment and concealed the bowls from the dog’s view, while the curtain served to temporarily conceal the actions of the experimenter from the dog’s view during the experimental procedures.

### 2.4. General Procedure

The test was based on a two-alternative forced choice, between two different quantities of food, in a ratio of 1 to 7. Prior to beginning the test, the dog and owner were taken into the room, and the dog was left free to explore and familiarize itself with the experimental setting and the experimenter for approximately 5 min. During this time, an experimenter explained the procedure to the owner. Then, the owner was invited to attach the leash to the dog and sit on the chair, and the experimental procedure began.

The experiment comprised a Pre-training phase, a Training phase, and a Test phase. All phases were composed of a number of consecutive trials following a similar procedure: the owner sat on the chair behind the curtain, holding the dog next to him/her. In a separate room, the experimenter baited the bowls with 7 food pieces (each being 1/4 of a ring of Frolic^®^, a commercial semi-humid dog food) of in one (S+) and 1 piece in the other (S−). Then, she entered the experimental room, placed the bowl(s) behind each plastic panel, walked towards the curtain and opened it, allowing the dog to see the two plastic panels. At that point, the experimenter walked behind the owner and placed a hand on the owner’s shoulder, which signalled that the dog could be released. The dog was allowed to reach only one of the two bowls, so as soon as the dog approached one bowl, the experimenter removed the other bowl, preventing the dog from eating its content. As soon as the dog ate the food, the selected bowl was also removed. Finally, the owner took the dog back to the starting position, and the curtain was again lowered, the experimenter went into the separate room to prepare the bowls for the next trials. If the dog did not make a choice within one minute, bowls were removed and the trial was considered null.

### 2.5. Pre-Training Phase

The aim of the Pre-training phase was to allow the dog to familiarize with the experimental procedure and experience that bowls in different location contained different amounts of food. This phase consisted of 6 trials, which followed the procedure described above, with the difference that only one food bowl was presented in each trial (S+ was presented on 3 trials, and S− on the other 3). In this phase, the food bowls were placed at the distance of 350 cm from the dog. For any given dog, S+ was always presented on the same side thorough the test, and S− on the opposite side. The side of presentation varied between subjects, and was counterbalanced within each of the four experimental groups. To be admitted to the training phase, dogs needed to promptly eat the food from the presented bowl in each of the 6 trials.

### 2.6. Training Phase

This phase was meant to teach dogs to choose the bowl containing the larger amount of food when both S+ and S− were presented simultaneously. Both S+ and S− bowls were placed at the same distance (P0, 350 cm from the dog). For each dog, S+ and S− were placed on the same side as in the Pre-training phase. A maximum of 30 trials were presented, and the criterion for passing this phase was to choose S+ in 6 consecutive trials. If a dog did not reach the learning criterion within the 30 trials, it was excluded from further testing. Before the test phase began, the owner was allowed to walk outdoors with her/his dog for 10 min.

### 2.7. Test Phase

The test phase was aimed at verifying the effect of increasing proximity of the smaller amount of food on dogs’ choice. The rationale for the test was that lower levels of impulsivity would result in dogs’ higher ability to choose the larger amount of food, despite the progressively higher proximity of the smaller food amount. The test phase consisted of 14 trials, which followed the general procedure, with the exception that, while S+ was always placed at the distance of 350 cm, the proximity of S− from dogs was systematically increased along a geometric progression. Specifically, there were three levels of increasing proximity: P1 (proximity increased by 40 cm compared to P0; distance of S− from the dog: 310 cm); P2 (proximity increased by 80 cm; 270 cm from the dog); P4 (proximity increased by 160 cm; 190 cm from the dog). Each of these three levels was presented three times among the fourteen trials; in the remaining 5 trials the distance of S+ and S− from the dog was the same (P0, 350 cm from the dog) as in the Training phase. The trials were randomly presented, with the constrain that S− could not be presented at the same distance in consecutive trials.

### 2.8. Data Collection and Analysis

All experiments were recorded by two ceiling-mounted cameras and coded with the Observer XT software (Ver.12.5, Noldus, Gröeningen, The Netherlands). In the Training and the test phases the dog’s choices were codified as S+, S−, or null.

The analysis of dogs’ choices in the Test phase aimed to provide an indication regarding the validity of the procedure. To this aim, the analysis was meant to verify that dogs’ ability to choose the larger food amount decreased as a function of the proximity of the smaller food amount. In addition, to obtain an indication about the specificity of the measure, the analysis was meant to exclude that the dogs’ performance reflected non-impulsivity related factors, such as different levels of motivation towards food, the dogs’ learning ability in acquiring the initial discrimination task, or the dogs’ training level. Finally, the analysis was aimed at highlighting possible differences in performance linked to the dogs’ age, sex and/or reproductive status, in accordance with associations between these factors and impulsivity reported in the literature, as an indication of the external validity of the procedure.

Training history was unevenly distributed across groups of different sex and reproductive status, making it impossible to include the variable in the model described below. To achieve a better distribution, we recoded the variable using the following two levels: “non professionally trained dogs”, which included untrained dogs, and dogs trained without support of a professional trainer, and “professionally trained dogs”, which included all other dogs. Prior to such recoding, we ascertained that training history had no main effect on dogs’ probability to make impulsive choices. To this aim, a Generalized Linear Mixed Model (GLMM) was used, which included the dog’s choice of S+ or S− as a binary dependent variable, the dogs’ ID as a random variable accounting for the repeated measurement within each dog, and training history as a four-level factor. As the GLMM revealed no significant main effects of training (*p* = 0.217), the variable was recoded which was used in the analysis described below.

To ascertain the specificity and external validity of our task, a GLMM was used, which included the dog’s choice of S+ or S− as a binary dependent variable, and the dogs’ ID as a random variable accounting for the repeated measurement within each dog. Separate models were run to investigate the effect of sex and reproductive status: one model was run on data collected from non-gonadectomized males and females and included the dog’s sex as fixed factor; the other two models were run on data collected respectively from females and males and included the reproductive status as a fixed factor. In addition to sex or reproductive status, the model included distance of S−, the dogs’ training history, and food motivation, as two-level fixed factors, and the dog’s age and number of trials to reach the learning criterion in the Training phase as covariates. First-order interactions between S− distance and each of the other fixed factors were also included in the model. A stepwise backwards elimination procedure was used to eliminate non-significant interactions. Post-hoc comparisons were run between factor levels when a significant effect was found for a factor, applying a sequential Bonferroni correction.

As the analysis revealed a significant effect of sex on dogs’ choices of S+ in the Test phase (see Results), a one-way ANOVA was performed to ascertain that there were no differences between dogs of different sex or reproductive status on their ability to acquire the initial Training phase, as measured by the number of errors made and in the number of trials needed to reach the learning criterion in such phase.

In order to further exclude that the dogs’ performance in the test reflected their learning ability in initial discrimination training, Pearson’s correlations coefficients were calculated between the number of trials needed to reach the learning criterion in the Training phase and the percentage of choices of S+, both across the entire test and at each different distance of S−.

Finally, as a further way to assess the relationship between the measure provided by the proximity test and other putative measures of impulsivity, Pearson’s correlation coefficients were calculated between the DIAS OQS, and the DIAS sub-scales scores, and the number of trials to reach criteria in the Training phase, the percentage of choices of S+ in the Test phase.

All analysis was run with SPSS (ver. 23, IMB, Armonk, NY, USA). A value of 0.05 was adopted as threshold for statistical significance.

## 3. Results

### 3.1. Pre-Training and Training Phases

All dogs involved in the study successfully passed the Pre-training and the Training phase. In the latter, dogs reached the training criterion with an average (± SD) of 10.8 ± 6.6 trials and made an average of 7.7 ± 3.0 choices of S+ and 3.1 ± 4.7 choices of S−. The mean ± SD number of errors (choices of S−) and of trials required to reach the learning criterion by dogs split by sex and reproductive status is reported in Table 2; the one-way ANOVA indicated that there were no differences between sexes in the number of errors (F = 1.09; *p* = 0.36) or trials required to reach the learning criterion in this phase (F = 0.56; *p* = 0.64). No null trials (i.e., a dog not approaching any of the two bowls) were observed by any dog. The Training phase was completed in an average of 14.6 ± 6.5 min (min: 5.5; max: 28.1).

### 3.2. Test Phase

Overall, dogs chose S+ in 75.8% of trials (mean N of trials ± SD: 3.79 ± 1.47 out of 5) when S− was presented at distance P0, 61.8% of trials (1.85 ± 1.15 out of 3) at P1, 35.4% (1.06 ± 1.25 out of 3) at P2, and 25.7% (0.77 ± 1.17 out of 3) at P4. The average of S+ choices for each of the four groups of different sex and reproductive status, at the different S− distances are summarized in Table 3. No null trials were observed in this phase. The Test phase was completed in an average of 15.4 ± 2.7 min (min:10.6; max: 24.4).

Table 4 summarizes the results of the three GLMM models, investigating the effect of S− distance, speed of acquisition of the Training phase, and dogs’ age and training history, food motivation and sex/reproductive status, on dog’s probability of choosing S+.

**Figure 2:**
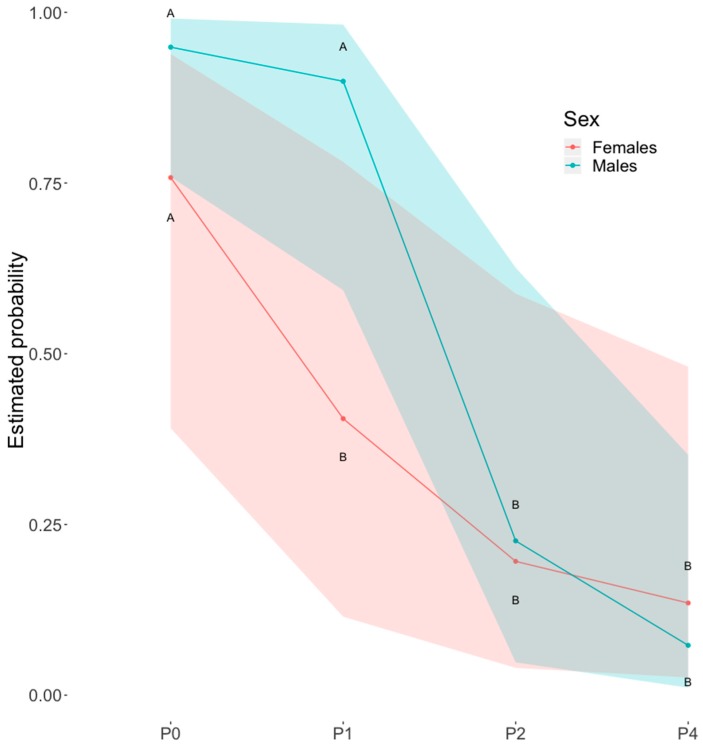
Choices of S− as a function of sex and distance. Generalized Linear Mixed Model (GLMM) mean estimates of the probability of choosing S+ as a function of the distance of S−, by intact male and female dogs. Shaded areas represent the lower and upper 95% confidence intervals. Different capital letters indicate significantly different probabilities between sexes and different levels of proximity of S− (*p* < 0.05) after sequential Bonferroni corrected post-hoc comparisons.

All models evidenced an effect of the distance of S− on dog’s probability of choosing S+, which generally decreased as S− was placed closer to the dog. When data from intact dogs were analysed, an effect of the interaction between the distance of S− and the dog’s sex was found (Figure 2). Post-hoc analysis revealed a significant difference between males and females in the probability of choosing S+ at distance P1 and, while in females the probability already decreased when S− was moved from P0 to P1, in males the first significant drop in probability was only observed when S− was moved from P1 to P2.

Models using data from the whole group of male dogs, and from the whole group of female dogs, revealed no effect of reproductive status on the probability of choosing S+ as a function of the distance of S−. None of the three models found any effect of the dog’s age, training history, food motivation, or speed of acquisition of the Training phase.

### 3.3. Correlations of Test Outcomes with Training Phase Performance and DIAS Scores

The DIAS questionnaire resulted in a mean ± SD of 0.51 ± 0.10 (range: 0.31–0.77) for the OQS, 0.48 ± 0.14 (0.28–0.88) for Factor 1, 0.45 ± 0.09 (0.28–0.68) for Factor 2 and 0.57 ± 0.09 (0.36–0.80) for Factor 3. Results of the correlation analysis between choices of S+ in the Test phase and both the speed of learning of the Training phase and the DIAS scores are reported in Table 5. No correlation was found between any of these variables. However, the number of trials to reach the criterion in the Training phase correlated positively with the DIAS OQS (Pearson’s correlation: 0.42, *p* < 0.01) and its score for Factor 1 (r = 0.44, *p* < 0.01) and Factor 2 (r = 0.40, *p* < 0.01), but not Factor 3 (r = 012, *p* = 0.44).

## 4. Discussion

In this study, we devised a behavioral test for the assessment of dogs’ tendency to make impulsive choices, which was conceived as a spatial implementation of the conventional delay discounting paradigm. All dogs who participated in the study successfully achieved the initial training, which required them to consistently select the larger of two food quantities presented at the same distance. In the subsequent test phase, as expected, dogs expressed a higher probability to choose the smaller amount of food, as the latter was positioned increasingly closer to the dog. The entire assessment procedure was completed in less than approximately 1 h. Overall, the findings represent a good indication of the feasibility of the paradigm, and its better suitability for the assessment of impulsive choices in dogs, compared with lengthier and harder-to-complete delay discounting tasks. A spatial discounting test analogous to the one presented in this study was independently developed and recently presented by Brady and collaborators [32]. This study also reports a high success rate, and an outcome which conformed to expectations (i.e., choices of the larger food amount dependent on the relative distance). Thus, in agreement with this study, we converge on this paradigm’s ease of application, which makes it a good candidate for the assessment of impulsivity in large dog samples.

Besides evaluating the feasibility of the procedure, we aimed at providing a first validation of the task as a measure of impulsive choices, by assessing its specificity and its external validity. To the first aim, we ascertained that dog’s performance in the spatial discounting task could not be explained by factors different from impulsivity. As our task was based on the acquisition of food, one of our first concerns was to exclude that the dogs’ performance did not reflect their motivation towards food rather than their impulsivity. The interplay between impulsive behaviour and motivation to obtain food is certainly a complex one [33]. In fact, while impulsivity and sensitivity towards food are independent traits, they interact to determine food-related behavioral outcomes in humans and rats [33,34]. To the best of our knowledge, the role of food motivation was seldom taken into account in dogs’ impulsivity studies. Brucks and collaborators [30] report that varying the quantity and the quality of food-rewards affects dogs’ ability to tolerate delays in a delay of gratification task. The same authors highlight the potential confounds represented by food motivational factors on impulsivity measures in dogs. Therefore, the finding that food motivation was not a significant predictor of the dogs’ performance in our task provides a first indication in favour of the tasks’ specificity. One caveat in the interpretation of these finding is that no dog was present with low levels of food motivation, restricting the validity of this claim to dogs with medium to high food motivation levels.

Past training was another factor that could potentially interfere with dogs’ performance in our tasks; for instance, dogs with experience of prolonged training may be more accustomed to sustained work and be less susceptible to mental fatigue, thereby performing better than untrained dogs in the test phase of our procedure. The finding that training had no effect in explaining dogs’ choices of the larger food amount was therefore another indication in favour of the tasks’ specificity as a measure of impulsivity. Importantly, while this result indicates that our assessment is unaffected by differences in training history, it does not negate that some forms of training may improve dogs’ ability to exert self-control. Recent findings suggest that specific forms of training can improve some measures of impulsive control, such as impulsive actions [26]. Moreover, deliberate training for self-control can lead to a generalized increased ability in different forms of impulsivity in humans, while extensive and specific training brings improvement in impulsive choice in animals [35]. However, data about the dogs’ training history is seldom reported in previous studies on dog impulsive choices, thus it is difficult to make conclusions about the role of such experience on this facet of impulsivity. The availability of an easily applicable procedure for the assessment of impulsive choices, like the one presented in the present study, will allow to study the role of specific training history on canine impulsivity.

A related finding was that the number of errors (S− choices) made by dogs before reaching the learning criterion in the training phase did not explain their choices in the test. Previous research highlighted how the task’s learning requirement may represent a confound in measures of dog impulsivity. In fact, the idea that alleged measures of impulsivity may actually reflect the dogs’ learning ability was presented as a potential explanation for the lack of consistency across tasks [29]. In view of such concerns, the finding that dogs’ performance in our assessment was not affected by the dogs’ ability to learn the initial tasks represents an important indication of specificity. Another concern that relates to the learning requirements of delay discounting tasks, is that the necessary initial training is often achieved only by a fraction of dogs, producing an inherent bias in the selection of dogs who undergo the actual assessment. This does not seem to apply to spatial discounting tasks, as the training phase was acquired by all dogs who participated in our study, as well as by nearly all those who took part in the task developed by Brady and collaborators [30].

As an indication of external validity, we investigated how dogs’ performance in our task was affected by age, sex, and reproductive status. Age had no effect on dogs’ probability to make impulsive choices. Considering the majority of our dogs were adults, the result is in line with human studies, where evidence indicates a stabilization of impulsive choice behavior after adolescence/young adulthood [16]. The performance of the dogs in our task showed a clear dimorphic pattern: females discounted more steeply, as their probability to choose the larger food amount decreased significantly as soon as the bowl with the smaller amount was moved closer to the dog. Many sex related behaviors have been described in dogs [36]. In the present study, analysis of sex differences was undertaken to provide an indication of the tasks’ goodness as a measure of impulsive behavior. In fact, our results conform to the what is reported in both humans and rodents, where steeper discount curves are generally found in females than in males [15]. No difference in performance was found between our intact and gonadectomized females. On the one hand this suggests that the main contribution to the observed sex difference is due to organizational effects of sex hormones, rather than by these hormone’s circulating levels. On the other hand, as our intact female dogs were in the anœtrous phase (based on the report of the owners on the date of their last manifestations of œstrous) it cannot be excluded that the performance of intact female dogs may have been different, had females been tested in other phases of the oestrous cycle, as seen in other species [37,38]. Our current data cannot elucidate the mechanisms underlying the observed differences, Thus, we cannot tell whether dopamine transmission is involved in these differences, as suggested for other species. To the best of our knowledge there is no data about sex differences or the role of ovarian hormones in dopaminergic transmission in dogs. However, it is worth noting that sex differences are consistently found in dogs’ spatial learning tasks [39,40,41], where dopamine plays a crucial role [42]. Regardless of the mechanism, our results indicate that the phenomenon our task is measuring is subject to the same biological influence seen in other species, providing an indication of the tasks’ external validity.

Finally, no correlation was found between our dogs’ performance in the test, and the score obtained by dogs in a putative assessment of impulsivity made through the DIAS questionnaire, either in terms of its overall score or the score of its subscales (calculated as described in the validation study by Wright and collaborators [18]). On the one hand, the finding clashes with the significant correlations between the DIAS score and the measures of impulsivity obtained in the spatial discounting task presented by Brady and collaborators [32], or in a delayed reward paradigm [19]. On the other hand, several other studies on dogs’ impulsive choices report no association with the DIAS score [25,29], or correlations in opposite directions than expected [30]. Although the reason of these discrepancies is not immediately clear, it must be considered that the DIAS was developed to assess impulsivity as a generic personality trait, rather than to pinpoint a specific facet of the phenomenon. As already highlighted by others [30], expressions of impulsivity are highly context specific and it is possible that the questionnaire and our task are assessing different facets of the same phenomenon. Alternatively, it is possible that they assess completely independent traits. In fact, our finding of a positive correlation between dogs’ speed of learning of the initial training phase and the questionnaire scores suggests that the latter reflects the dogs’ learning ability rather than their impulsivity. Moreover, questionnaires are based on indirect evaluations of the animals’ behavior made by their owners, which incorporates a considerable degree of subjectivity in the assessment. Such individual variability could be further amplified by cultural differences, and translation-related nuances. In fact, while significant correlations between the DIAS and impulsivity measures were reported by studies conducted in the UK, the opposite was generally true for studies made in non-Anglo-Saxon countries, either using the original English version (e.g., [25]) or a translated version of the questionnaire [29,30], as in the current one.

## 5. Conclusions

In this study we presented a spatial discount task, aimed at assessing impulsive choices in dogs. A similar task was independently developed by Brady and collaborators [32] at around the same time. Both studies converge on the ease of application of the task, which advocates the procedure as a good candidate for larger-scale studies on impulsivity. We ascertained the lack of effect of several factors which may have interfered with the dogs’ measure, thereby providing indications of the procedure’s specificity. In addition, we provided indications about its external validity by showing a susceptibility of the assessment to sex differences, similar to those already observed in humans and rodents. Overall, the task seems to be promising as a valid, easily applicable procedure for the assessment of impulsive choices.

However, although these findings, together with those of Brady and collaborators [32], provide indications about the goodness of this assessment, other steps would be needed to provide conclusive evidence of its validity, as well as to fine-tune the procedure. For instance, it would be important to determine how the present assessment relates to the outcome of other procedures, that are assumed to measure other facets of impulsivity, such as tasks assessing dogs’ tendency to express impulsive actions. Moreover, in view of a potential application in large-scale or cross-cultural studies, it would be important to extend the assessment to larger representation of size and age than those included in this study, as well as to ascertain the reproducibility of the assessment across different laboratories. Considering the ease of administration of the procedure, it is foreseeable that the same would be applied as a screening/selection tool in clinical (e.g., for the identification of pathological impulsivity) or other professional contexts (e.g., for the selection of dogs to be trained for specific activities); to this aim, evaluation of the applicability of the procedure in non-experimental settings and of its predictive validity for expected outcomes, would be required. Finally, considering the known interplay between training and impulsivity, the procedure could be used to assess the efficacy of specific forms of training, including its applications as a therapeutic intervention, in reducing impulsive behavior.

## Figures and Tables

**Table 1:** Distribution of categories of Training history and Food motivation within groups of dogs of different sex and reproductive status.

Sex/Reproductive Status	Training History				Food Motivation		
	No	Home	Obedience	Work	Low	Medium	High
Intact males	1	5	3	3	0	7	5
Intact females	0	7	1	4	0	5	7
Orchiectomized males	2	5	4	1	0	5	7
Ovariectomized females	0	6	2	4	0	5	7

**Table 2:** Mean ± SD number of errors (choices of S−) and trials required to reach the learning criterion (TTC) in the Training phase by dogs of different sex and reproductive status.

Parameter	Intact Males	Intact Females	Orchiectomized Males	Ovariectomized Females
Errors	1.2 ± 1.6	4.4 ± 6.9	3.1 ± 4.0	3.7 ± 4.3
TTC	8.7 ± 3.6	11.7 ± 8.4	11.3 ± 7.3	11.6 ± 6.6

**Table 3:** Mean ± SD number of S+ choices in the Test phase by dogs of different sex and reproductive status. In brackets: mean ± SD percentage of S+ choices on the total number of trials for each distance (i.e., 5 for P0, 3 for P1, P2, and P4).

Distance	Intact Males	Intact Females	Orchiectomized Males	Ovariectomized Females
**P0**	4.3 ± 1.4 (85 ± 27%)	3.5± 1.6 (70 ± 32%)	3.4 ± 1.6 (68 ± 31%)	4.0 ± 1.4 (80 ± 27%)
**P1**	2.3 ± 0.9 (78 ± 30%)	1.4 ± 1.3 (47 ± 43%)	1.8 ± 1.0 (61 ± 34%)	1.8 ± 1.3 (61 ± 42%)
**P2**	1.1 ± 1.2 (36 ± 39%)	1.1 ± 1.2 (36 ± 39%)	1.0 ± 1.4 (33 ± 45%)	1.1 ± 1.4 (36 ± 48%)
**P4**	0.5 ± 1.0 (17 ± 33%)	0.8 ± 1.0 (28 ± 34%)	0.8 ± 1.2 (25 ± 41%)	1.0 ± 1.5 (33 ± 49%)
**Overall**	8.25 ± 3.2 (58 ± 23%)	6.8 ± 4.4 (48 ± 31%)	7.1 ± 4.4 (50 ± 30%)	7.9 ± 4.6 (56 ± 33%)

**Table 4:** Results of the Generalized Linear Mixed Models investigating the effect of the distance of the bowl with the smaller amount of food, the dog’s sex or reproductive status (investigated in separate models, and with different data subsets), age, food motivation, type of training received, and number of trials needed to reach the learning criterion in the Training phase (TTC). Only significant first-order interactions between distance and other factors are reported. IF = intact females, IM = intact males, OF = ovariectomized females, OM = orchiectomized males. Subscript numbers indicate the numerator and denominator degrees of freedom, respectively.

Factor	Experimental Groups Which Data Were Analysed in the Model		
	IF and IM	OF and IF	OM and IM
Distance	F_3,303_ = 5.17; *p* = 0.002	F_3,303_ = 3.26; *p* = 0.022	F_3,303_ = 2.93; *p* = 0.034
Sex	F_1,303_ = 0.061; *p* = 0.805	-	-
Reproductive status	-	F_1,303_ = 0.77; *p* = 0.380	F_1,303_ = 0.61; *p* = 0.433
Age	F_1,303_ = 2.67; *p* = 0.197	F_1,303_ = 2.10; *p* = 0.149	F_1,303_ = 0.43; *p* = 0.512
TTC	F_1,303_ = 0.55; *p* = 0.457	F_1,303_ = 1.42; *p* = 0.234	F_1,303_ = 0.13; *p* = 0.723
Food motivation	F_1,303_ = 0.88; *p* = 0.353	F_1,303_ = 0.92; *p* = 0.337	F_1,303_ = 1.33; *p* = 0.250
Training history	F_1,303_ = 1.50; *p* = 0.222	F_1,303_ = 0.030; *p* = 0.863	F_1,303_ = 0.71; *p* = 0.401
S− distance × Sex	F_3,303_ = 2.70; *p* = 0.045	-	-

**Table 5:** Pearson’s correlations coefficients between the percentage of choices of S+ in the test phase, both at different S− distances (P0 = 350 cm, P1 = 310 cm, P2 = 270 cm, P4 = 190 cm) and across the whole test, and the number of trials needed to reach the learning criterion in the Training phase (TTC), the DIAS overall score (OQS), the score of the DIAS’ Factor 1, Factor 2 and Factor 3.

	P0	P1	P2	P4	Overall
TTC	0.026	−0.063	−0.160	−0.255	−0.108
DIAS OQS	0.243	−0.167	−0.074	−0.025	0.010
DIAS Factor 1	0.295	−0.135	−0.017	0.040	0.075
DIAS Factor 2	−0.216	−0.140	−0.142	−0.203	−0.221
DIAS Factor 3	−0.220	−0.035	−0.100	−0.011	0.036
